# Occurrence of Nodular Lymphocyte-Predominant Hodgkin Lymphoma in Hermansky-Pudlak Type 2 Syndrome Is Associated to Natural Killer and Natural Killer T Cell Defects

**DOI:** 10.1371/journal.pone.0080131

**Published:** 2013-11-26

**Authors:** Luisa Lorenzi, Giovanna Tabellini, William Vermi, Daniele Moratto, Fulvio Porta, Lucia D. Notarangelo, Ornella Patrizi, Silvano Sozzani, Genevieve de Saint Basile, Sylvain Latour, David Pace, Silvia Lonardi, Fabio Facchetti, Raffaele Badolato, Silvia Parolini

**Affiliations:** 1 Department of Molecular and Translational Medicine, Section of Pathology, University of Brescia, Brescia, Italy; 2 Department of Molecular and Translational Medicine, Section of Experimental Oncology and Immunology, University of Brescia, Brescia, Italy; 3 Department of Pathology and Immunology, Washington University School of Medicine, St. Louis, Missouri, United States of America; 4 Department of Clinical and Experimental Sciences, Institute of Molecular Medicine Angelo Nocivelli, University of Brescia, Brescia, Italy; 5 Oncology-Haematology and BMT Unit, Ospedale dei Bambini, Spedali Civili, Brescia, Italy; 6 INSERM, U768; University Paris Sorbone, France; 7 Centre d'étude des deficits immunitaires, AP-HP, Necker-Enfants Malades Hospital, Paris, France; 8 Department of Pediatrics, Mater Dei Hospital, Tal-Qroqq, Msida, Malta; Karolinska Institutet, Sweden

## Abstract

Hermansky Pudlak type 2 syndrome (HPS2) is a rare autosomal recessive primary immune deficiency caused by mutations on β3A gene (AP3B1 gene). The defect results in the impairment of the adaptor protein 3 (AP-3) complex, responsible for protein sorting to secretory lysosomes leading to oculo-cutaneous albinism, bleeding disorders and immunodeficiency. We have studied peripheral blood and lymph node biopsies from two siblings affected by HPS2. Lymph node histology showed a nodular lymphocyte predominance type Hodgkin lymphoma (NLPHL) in both HPS2 siblings. By immunohistochemistry, CD8 T-cells from HPS2 NLPHL contained an increased amount of perforin (Prf) + suggesting a defect in the release of this granules-associated protein. By analyzing peripheral blood immune cells we found a significant reduction of circulating NKT cells and of CD56^bright^CD16^−^ Natural Killer (NK) cells subset. Functionally, NK cells were defective in their cytotoxic activity against tumor cell lines including Hodgkin Lymphoma as well as in IFN-γ production. This defect was associated with increased baseline level of CD107a and CD63 at the surface level of unstimulated and IL-2-activated NK cells. In summary, these results suggest that a combined and profound defect of innate and adaptive effector cells might explain the susceptibility to infections and lymphoma in these HPS2 patients.

## Introduction

The role of the immune system in cancer surveillance has been characterized in detail at the cellular and molecular level [1], [2]. Lymphoproliferative disorders (LPD) are among the most frequent spontaneous neoplasms arising in immunodeficient mice [3]. In humans, the risk of developing LPD is significantly increased in primary and secondary immunodeficiencies. In particular, primary immune deficiency (PID) patients might develop a wide array of LPD, sharing features such as extra-nodal involvement, predominance of high-grade B-cell neoplasm and frequent association with Epstein Barr Virus (EBV) infection [4]. Although Hodgkin Lymphoma (HL) has been reported in patients with secondary immune deficiencies, such as iatrogenic immunosuppression and HIV infection [5], it is rarely observed in PID. Cases of classical HL have been reported in patients with Hyper-IgM (HIGM) syndrome, Common Variable Immunodeficiency (CVID), Hyper-IgE syndrome (HIES) and Wiskott Aldrich Syndrome (WAS) [5], [6]. On the contrary, nodular lymphocyte predominance HL (NLPHL) was reported only in association with autoimmune lymphoproliferative syndrome (ALPS) [7], [8].

Hermansky Pudlak type 2 syndrome (HPS2) is a rare autosomal recessive disease characterized by oculo-cutaneous albinism, bleeding disorders and immunodeficiency [9], [10]. The disease is caused by mutations on the β3A gene (AP3B1) encoding for the β3A subunit of the adaptor protein 3 (AP-3) complex. This heterotetrameric complex is an ubiquitously expressed cytosolic protein, that is essential for secretory lysosomes formation in melanocytes, platelets, neutrophils, cytotoxic T cells (CTL), and Natural Killer (NK) cells. In the immune system, absence of AP-3 leads to reduced intracellular content of neutrophil elastase and consequently to neutropenia. Likewise, defects in cytolytic activity have been observed in vitro in NK cells and CTL of HPS2 patients [11], [12]. NK cells are essential for tumor surveillance and defense against virally infected cells [13].

Natural Killer T (NKT) cells are a distinct lymphocyte subset characterized by expression of CD3 and CD56. These cells have been defined as an innate-like lymphocyte population that express an invariant TCR made of the Ja18-Vα24 and Vβ11 rearrangements specific for glycosphingolipids presented by the non-classical MHC Class-I molecule CD1d. iNKT cells display important immune regulatory functions [14]. Compelling evidence indicate that iNKT cells might have an important role in tumor surveillance. iNKT cells exhibit direct anti-tumor activity and enhance the cytotoxic activities of NK and CD8+ T cells. Significantly, a decrease in iNKT cells in the peripheral blood or tissues is observed in patients with advanced forms of cancer [15].

In this study, we have investigated the immune functions of NK and NK-T cells in in two siblings affected by HPS2.

## Materials and Methods

### Patients

The investigation was conducted according to the principles expressed in the Declaration of Helsinki and approved by the local ethic committees. All subjects, caretakers, or guardians on the behalf of the minors/children participant gave their written informed consent to participate in the study as approved by the local ethic committee at Spedali civili, Brescia. Written informed consent for the publication of case history from the next of kin, caretakers, or guardians on the behalf of the minors/children participants involved in your study was obtained.

Born from unrelated parents, Patient 1 (Pt1) and Patient 2 (Pt2) were diagnosed with HPS2 at the age of 7 and 4 years respectively at Spedali civili (Brescia, Italy) as previously described [12]. Patient 3 (Pt3) was diagnosed at the age of 7 months at Mater Dei Hospital, Tal-Qroqq, Msida, Malta. Partial oculocutaneous albinism was observed in the patients at birth.

At the age of 10 Pt1 presented with asymptomatic left mandibular lymphadenopathy and Positron Emission Tomography (PET) showed bilateral involvement of laterocervical lymph nodes. At the age of 8 years, a retroperitoneal mass was incidentally detected in Pt2. Stage IIA and Stage IIIA NLPHL were diagnosed respectively; complete remission was achieved in both patients upon treatment with the AIEOP MH-2004 chemotherapeutic protocol; after 53 and 37 months from diagnosis respectively both patients are free of disease.

Pt 3 is a 6 year old Maltese girl who was clinically suspected to have HPS2 at 7 months of age. Aged 2 months, she presented with horizontal nystagmus which was found to be secondary to *albinoid fundi*. In addition, she was noted to be generally hypopigmented, had severe neutropenia, low serum IgM and prolonged in vitro bleeding time. HPS2 was confirmed by mutational analysis of the AP3B1 gene (g.180117-180740 del). Subsequently, she was diagnosed with bilateral developmental dysplasia of the hips necessitating open reduction, pectus excavatum and asymptomatic ventricular ectopics. At the age of 5 she acquired primary EBV infection from which she recovered completely.

Whole blood was collected from HPS2 patients and from healthy donors in BD Vacutainer Plus plastic whole blood tubes (BD Bioscience, Franklin Lake, NJ, USA) and then used for cells purification. Fresh blood was available for a limited number of experiments for Pt3.

### Histology and Immunohistochemistry

Tumor specimens were represented by formalin fixed and paraffin embedded lymph nodes from the two patients and five cases of NLPHL from patients without history of immunodeficiency (4 males, 1 female, from 18 to 73 years old). Paraffin sections were used for immunohistochemistry to detect the following antigens: CD20 (clone L26, Dako, Glostrup, Denmark), PAX5 (clone 24/PAX-5, BD Biosciences, San Josè, CA, USA), Bcl6 (clone P1F6, Novocastra Laboratories, Newcastle upon Tyne, UK), BOB.1 (rabbit polyclonal, Santa Cruz Biotechnology Inc, Santa Cruz, CA, USA), OCT-2 (rabbit polyclonal, Santa Cruz Biotechnology), CD30 (clone BerH2, Dako), CD15 (clone MMA, Thermo Scientific, Fremont, CA, USA), CD3 (clone SP7 Thermo Scientific), CD8 (clone C8/144B, Dako), PD1 (clone NAT 105/e3J, kindly provided by Dr Teresa Marafioti, Oxford), CD57 (clone NK-1, Invitrogen Corporation, Carlsbad, CA, USA), CD56 (mouse IgG1, clone Ab-2, Thermo Scientific), Perforin (clone 5B10, Novocastra Laboratories), CD23 (clone 1B12, Thermo Scientific), CD21 (clone 2G9, Thermo Scientific), CD163 (clone 10D6, Thermo Scientific), CD68R (clone PG-M1, Dako).

The immunoreaction was revealed using Envision MR (Dako) or NovoLink Polymer (Novocastra Laboratories) peroxidase-conjugated polymers, followed by diaminobenzidine as chromogen and hematoxylin as counterstain. Double immunostains for perforin (Prf), CD8 and CD56 and for CD56 and CD3 were performed as previously reported [16]. The second immune reaction was revealed using Mach4 AP (Biocare Medical, Concord, CA, USA) followed by Ferangi Blue as chromogen (Biocare Medical). Detection of Epstein-Barr virus (EBV) was performed by immunohistochemistry, using antibody against LMP1 (clone CS1, Novocastra Laboratories) and by in situ hybridization of EBV-encoded RNA (EBV/EBER), (PNA ISH Detection Kit, Dako).

For cell counting digital images taken with DP-70 Olympus digital camera mounted on Olympus BX60 microscope were processed by Analysis Image Processing software (Olympus). The number of Prf positive cells were evaluated on five high power field (HPF, corresponding to 1.8 mm^2^) for each case and the values were expressed as mean of positive cells +/− SEM per HPF. The student's t test was used for statistical analysis and considered significant with values of p<0.05.

### PBMC and NK cells purification and culture

Peripheral blood mononuclear cells (PBMC) derived from patients and healthy donors seen for minor trauma were obtained from heparinized blood by density gradient centrifugation over Ficoll (Sigma, St. Louis, MO). PBMC were resuspended in RPMI 1640 medium, supplemented with 2 mM glutamine, 50 µg/ml penicillin, 50 µg/ml streptomycin and 10% heat-inactivated FCS (Fetal Calf Serum, Sigma, St. Louis, MO). Peripheral blood samples were collected from patients before the development of NLPHL. Peripheral blood collected from three different age-matched healthy donors were used in every experimental assays.

NK cells were purified by NK Cell Separation Cocktails (Rosette Sep, Stem Cell Technologies Inc, Vancouver, BC). The purity of NK cells was >96% as assessed by flow cytometric analysis with a mixture of CD56-PC5 and CD3-FITC antibodies. CD3 contamination in purified NK cells was <1%. Purified NK cells were cultured on irradiated feeder cells in the presence of 100 U/ml IL-2 (Proleukin, Chiron Corp., Emeryville, USA) and 1.5 ng/ml PHA (Gibco Ltd, Paisley, Scotland) in order to obtain polyclonal NK cell populations.

### Flow cytofluorimetric analysis

The fine characterization of surface markers of resting NK cells was performed using the following mAbs produced in our laboratory or kindly provided by A. Moretta (DIMES, University of Genoa): BAB281 and KL247 (IgG1 and IgM respectively anti-NKp46), AZ20 and F252 (IgG1 and IgM, respectively, anti-NKp30), AZ140 and KS38 (IgG1 and IgM respectively anti-NKp44), ON72 and BAT221, (IgG1 anti-NKG2D), c127 and SUS142 (IgG1 and G2b respectively, anti-CD16), c218 and FS280 (IgG1 and IgG2a, respectively, anti-CD56), PP35 (IgG1, anti-CD244), A6/136 (IgM, anti-HLA class I), 3C8 (IgM, anti-CD63), XA147 (IgM anti-CD57).

A mixture of PC5-conjugated anti-CD56 mAb and FITC-conjugated anti-CD3 mAb (Beckman Coulter, Immunotech, Marseille, France), PE-conjugated anti-CD107a mAb (Becton Dickinson- Biosciences, Pharmingen, CA, USA), purified anti-CXCR1 (Santa Cruz Biotechnologies, Santa Cruz, CA) and purified anti-CCR7 and PC5-conjugated anti-CD62L (R&D systems, Minneapolis MN USA) were purchased for further cytofluorimetric analysis. CD107a expression was evaluated without tumor target cells on freshly isolated and polyclonal IL-2 activated NK cells staining with anti–CD3-FITC and anti–CD56-PC5 mAbs for 30 minutes, and afterward incubated with 4 µl anti-CD107a-PE for 1 hour.

For detection of IFN-γ production, polyclonal IL-2-activated NK cells were stimulated with PHA (1.5 ng/ml) or K562 (E/T ratio: 1∶1) for 3 hours at 37°C. NK cells were washed, fixed and permeabilized with Fix and Perm Solution (BD Biosciences) for 20 minutes and then labeled for 1 hour with 50 µg/ml anti-IFN-γ-PE (Becton Dickinson-Biosciences, Pharmingen, CA, USA).

Purified PBMC (10^6^ cells) from peripheral blood samples were used to evaluate the proportion of iNKT cells among the lymphocyte population for each individual tested. Identification of iNKT cells was obtained co-staining a commercial FITC-conjugated antibody directed against the CDR3 region of the invariant TCRα chain (Vα24-JαQ) (BD Biosciences) with fluorochrome-conjugated anti-CD3, anti-CD16 and anti-CD56 mAbs (BD Biosciences) or with the combination of FITC-conjugated antibody against TCR Vα24 (BD Biosciences) and PE-conjugated antibody against TCR Vβ11 (BD Biosciences) with APC- conjugated anti-CD3 for 20 minutes at RT. An alternative protocol required staining for 30 minutes with a 1∶200 dilution of PE-conjugated PBS-57 loaded CD1d tetramer (obtained from the NIH Tetramer Core Facility at Emory, Atlanta), used as a specific iNKT cell marker. At least 250,000 PBMC were acquired for each test and the proportion of iNKT cells was calculated after selection of CD3^+^ lymphocytes that coexpressed the specific NK and iNKT cell markers. Non-specific staining was evaluated using an appropriate isotype control mAb (BD Biosciences) or the unloaded CD1d tetramer. All cell acquisitions were performed on a FACSCalibur flow cytometer (BD) and data analyzed using the Cell Quest software (BD) or the FlowJo software version 8.8.6.

### Cell Lines and Cytotoxicity Assays

NK cells that had been exposed to IL-2 were tested for cytolytic activity against various NK-susceptible tumor target cells, including: Hodgkin's-derived cell lines L540 and L428, human melanoma FO-1 and M14, murine mastocytoma P815, EBV-lymphoblastoid cell lines HLA^−^ LCL 721.221, EBV-lymphoblastoid cell lines HLA^+^ ALINA, Burkitt's lymphoma Daudi and Raji, ovarian carcinoma IGROV, human glioblastoma A172, erythroleukemia K562, kidney carcinoma SKNEP1, and allogeneic PHA blasts [17]–[22]. These cell lines were selected on the basis of their expression of specific ligands for activating NK receptors. In particular, FO-1, RAJI express ligands for NKG2D and NKp30 while M14, P815, B-EBV lymphoblastoid cell lines mainly express the ligands for NKp46 [23], [24].

All these tumor cell lines were tested in a 4-h ^51^Cr release assay as previously described [25] with 5•10^3^cells/well at a final ratio of 1∶10 with polyclonal activated NK cells. L540 and L428 were kindly provided by Marco Cassatella (University of Verona, Italy), all other target cell lines were kindly provided by Alessandro Moretta (University of Genoa, Italy).

Effector NK cells were incubated either in the absence or in the presence of specific mAbs, IgM isotype, anti-HLA class I and/or anti-NK cell receptors.

All the *in vitro* experiments were performed three times for both patients, each time with three different healthy controls. All the values obtained from the patients' and control's groups were pulled together and analyzed using the Mann-Whitney test. The results of the statistical analyses between the two groups are shown in the Figures, with asterisks indicating p values <0.01.

## Results

### Development of Nodular Lymphocyte-Predominant Hodgkin Lymphoma in two HSP2 patients

**Figure 1:**
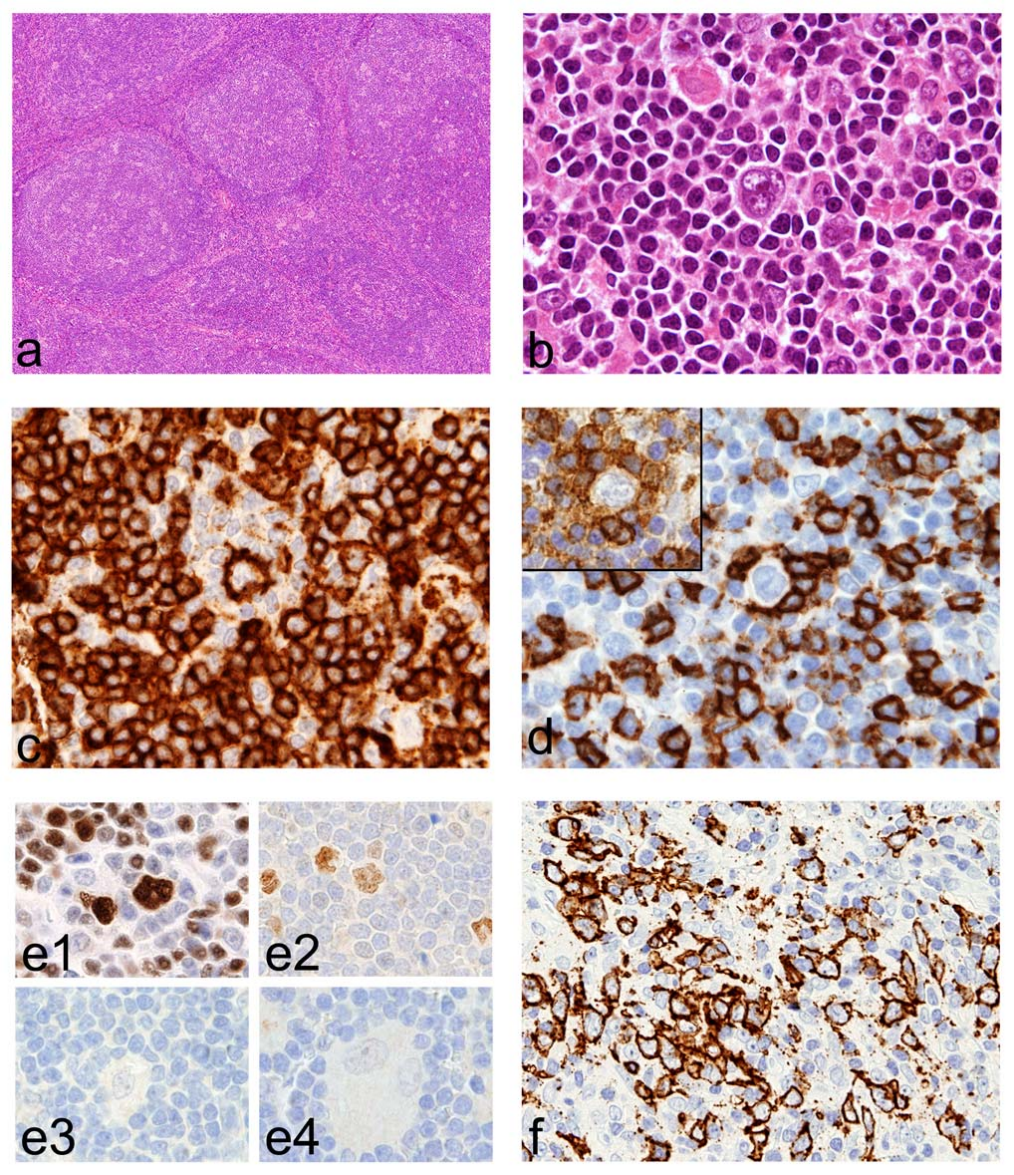
Histology of NLPHL in HPS2 patients. Lymph node biopsy from Pt1 show multiple large nodules (a) containing LP cells (b) that express CD20 (c) and are surrounded by rosettes of CD57^+^ (d), PD-1^+^ (d inset) T-cells. LP cells are positive for OCT2 (e1) and Bcl6 (e2) and negative for CD15 (e3) and CD30 (e4). In Pt2, an area with accumulation of numerous, partially confluent, large CD20^+^ B cells, suggests histological progression (f). For immunohistochemistry, sections are counterstained with Meyer's haematoxylin and secondary antibodies revealed with DAB. Original magnification: 40×(a); 200×(f); 600×(b, c, d, e).

Pt1 and Pt2 developed NLPHL at the age of ten and eight, respectively (see Material and Methods section for details). The histological features in Pt1 and Pt2 lymph nodes were similar. Lymph nodes showed a diffusely effaced architecture (Figure 1 a), with partially confluent macro-nodules mainly composed by small mature lymphocytes; a large number of epithelioid histiocytes were also present within the nodules. Atypical cells with classical cytologic and phenotypic features of lymphocyte predominant (LP) cells (strong expression of CD20, Pax5, Bcl6, OCT2 and BOB.1, negativity for CD15 and CD30) were found within the nodules (Figure 1 b, c, e). These nodules were mainly composed by CD20^+^ small B-cells (Figure 1 c) and contained an expanded meshwork of CD21^+^CD23^+^ follicular dendritic cells (not shown). Rosettes of CD3^+^CD57^+^PD1^+^ T cells surrounding LP cells were commonly observed (Figure 1 d). The intranodular epithelioid histiocytes reacted for CD163 and CD68 (not shown). In Pt2, an extra-capsular area of the lymph node showed accumulation of numerous confluent large CD20^+^ B cells on a background of T-lymphocytes, suggesting an histological progression (Figure 1 f). No EBV-infection was detected by immunohistochemistry and *in situ* hybridization.

**Figure 2:**
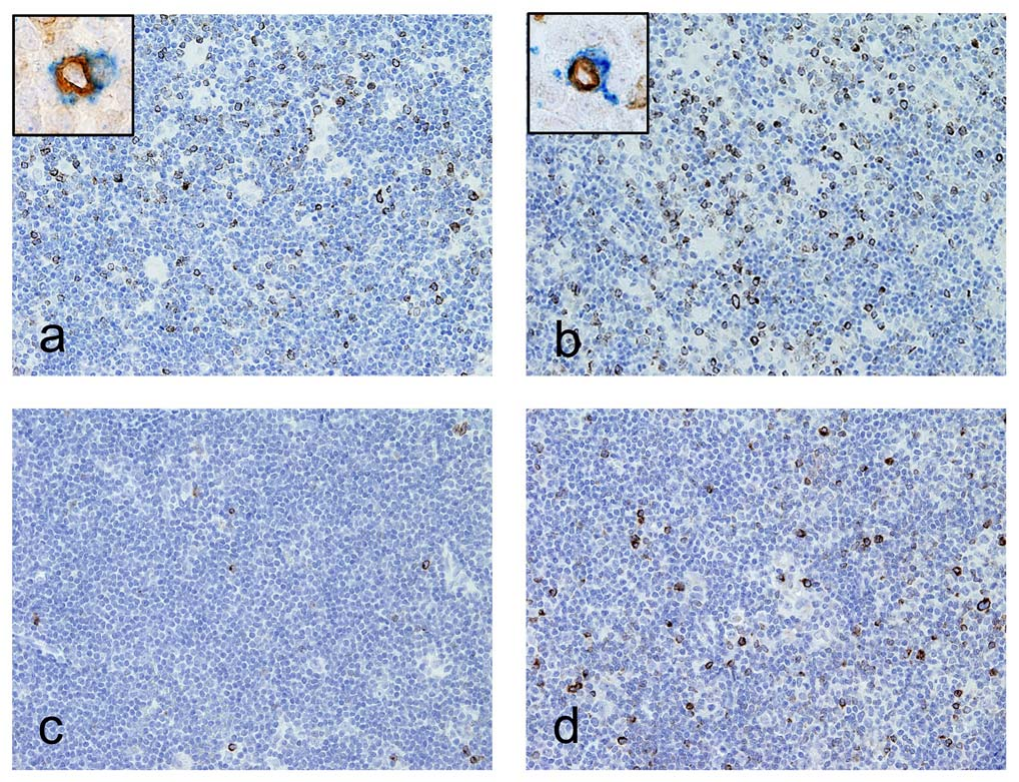
Prf-positive cells in NLPHL. Lymph node sections are from Pt1 (a), Pt2 (b) and controls (c and d), stained for anti-Prf (a-d; brown) and anti-CD8 (inserts blue). An increased number of Prf+ CTL co-expressing CD8 (insert) are observed in NLPHL nodules from HPS2 patients compared to controls. Sections are counterstained with Meyer's haematoxylin and secondary antibodies revealed with DAB (Prf) or Ferangi blue (CD8). Original magnification: 200×(a–d) and 1000×(inserts).

We analyzed the effector cell populations in the tumor microenvironment of NLPHL by immunohistochemistry. As observed in other cases of NLPHL, diagnosed in other subjects, host immune cells in NLPHL from HPS2 patients were mainly represented by numerous CD8^+^ T-cells and very rare CD56^+^CD3^−^ NK cells (data not shown). Conversely, we found that the number of cells expressing perforin (Prf^+^) in NLPHL from HPS2 patients was significantly higher compared to other cases of NLPHL (Pt1: 48±15, Pt2 82±14; NLPHL-controls: 20±6; p = 0.005) (Figure 2). The large majority of Prf^+^ cells were located within the tumor nodules and co-expressed CD8 thus corresponding to NLPHL-associated CTL. In addition, double stains for Prf and CD8 (Figure 2 a and b inserts) showed a significant increase in the fraction of Prf^+^ CD8^+^ CTL in NLPHL from HPS2 patients (Pt1: 54%±5%, Pt2: 55%±12%; NLPHL-controls: 27%±11%; p = 0.03) suggesting increased Prf retention in HPS2 CTLs. However, analysis of intracellular perforin content by flow cytometry of CD3+CD8+ T cells derived from patients Pt1 and Pt2 PBMCs showed that intracellular staining of perforin was comparable to that of healthy donors (data not shown).

### NK cells from HPS2 patients show a reduction of the CD56^bright^ subset and have an altered expression of the degranulation markers CD63 and CD107a

**Figure 3:**
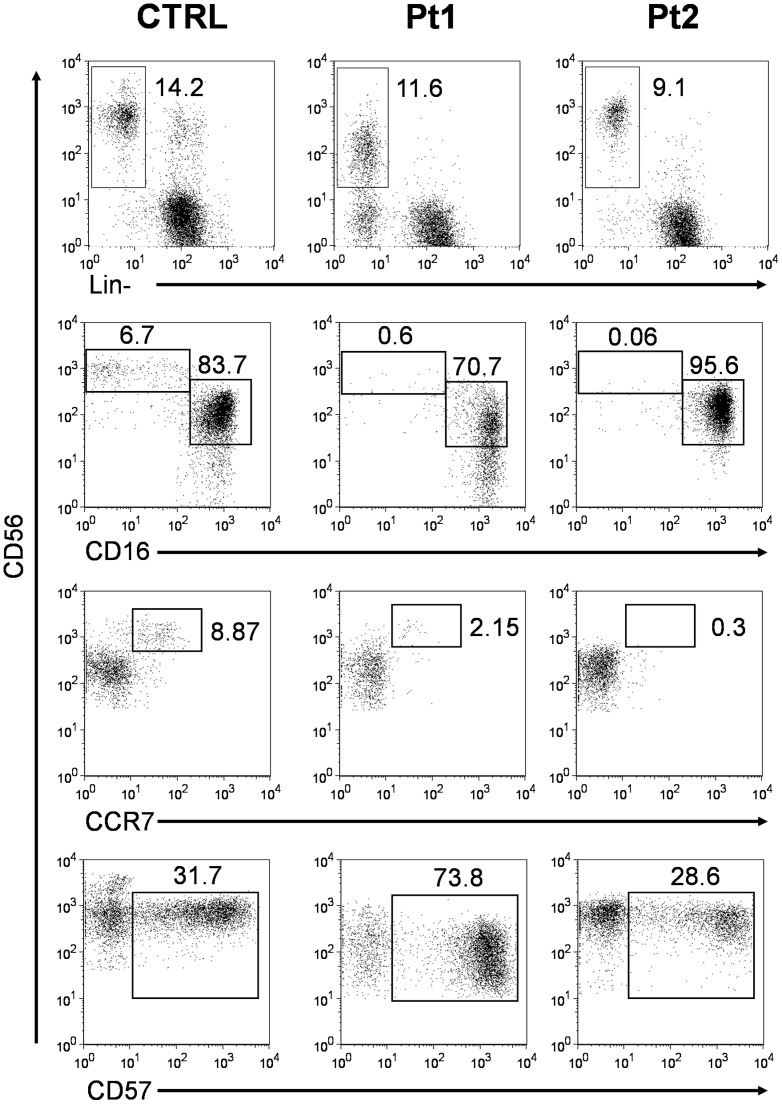
Analysis of the CD16, CCR7, and CD57 surface expression on CD56^+^ NK cells. Flow cytometry relative quantification of CD56^+^ Lin^−^ NK cells (first panels) and of CD16 (second panels), CCR7 (third panels), and CD57 expression (lower panels) on CD56^+^ gated NK cells. HPS2 patients' (Pt1 and Pt2) data are representative of one of the four separate evaluations performed on peripheral blood lymphocytes in a two-year period and are compared with a representative normal subject (CTRL) of the healthy donor group.

We hypothesized that the susceptibility to this rare form of lymphoma in HPS2 patients might be related to defective immune surveillance. Therefore, we analyzed specific markers of maturation and degranulation on NK cells by flow cytometry. In both HPS2 patients we observed a striking reduction of the CD56^bright^/CD16^−^/CCR7^+^ NK cell subset and an important increase of CD56^dim^ NK cells (Figure 3). Specifically, from four separate evaluations in a two-year period, the average percentage of CD56^bright^ cells on total NK cells was 1.4±0.9% and 0.15±0.11% for Pt1 and Pt2 respectively, compared to a median of 8.6% (range 1.8–23.2) calculated in 22 age-matched healthy subjects. Recent studies suggest that CD57, CD16 and KIR expression by CD56^dim^ NK cells is associated with phenotypical and functional features of highly mature and terminally differentiated NK cells [26]–[29]. Analysis of co-expression of CD16, CD57 and KIR in NK cells from HPS2 patients showed a significant depletion of CD56^bright^/CD16^−^ and of CD56^bright^/CCR7^+^ subsets (Figure 3 and data not shown) that are usually seen as NK cells with potent immunoregulatory function, but reduced cytolitic activity, suggesting that circulating NK cells display a terminally differentiated phenotype. This is especially evident in Pt1 that shows an increase of CD56^+^/CD57^+^ NK cells. The expansion of the CD56^+^/CD57^+^ subset might be related to the numerous episodes of viral infections that have been observed in Pt1, suggesting that recurring exposures of HPS2 patients to viral pathogens might have led to depletion of the CD56^bright^/CD16^−^/CCR7^+^ subset and, secondarily, to expansion of the of CD56^+^/CD57^+^ memory NK subset [28]. We also analyzed expanded IL-2 activated NK cells and we noted, in both patients, peculiar expansion of KIRs+/NKG2A- NK cell clones (data not shown) when compared with healthy donors.

Next, we studied the expression of lysosomal and degranulation markers on resting and IL2-activated NK cells. Previous studies by our group and others [12], [30], [31] reported an abnormal expression of the lysosomal marker CD63 on cell membrane of neutrophils and CD8+ T-cells from the same HPS2 patients [12], [31]. Flow cytometry analysis showed that a large fraction of unstimulated NK cells (25% for Pt1 and 30% for Pt2) and IL-2 activated NK cells in HPS2 (78–96% in both patients) expressed CD63 as compared to the control group (0.1–1% and 5–17% in unstimulated and IL-2 activated NK cells, respectively) (data not shown).

**Figure 4:**
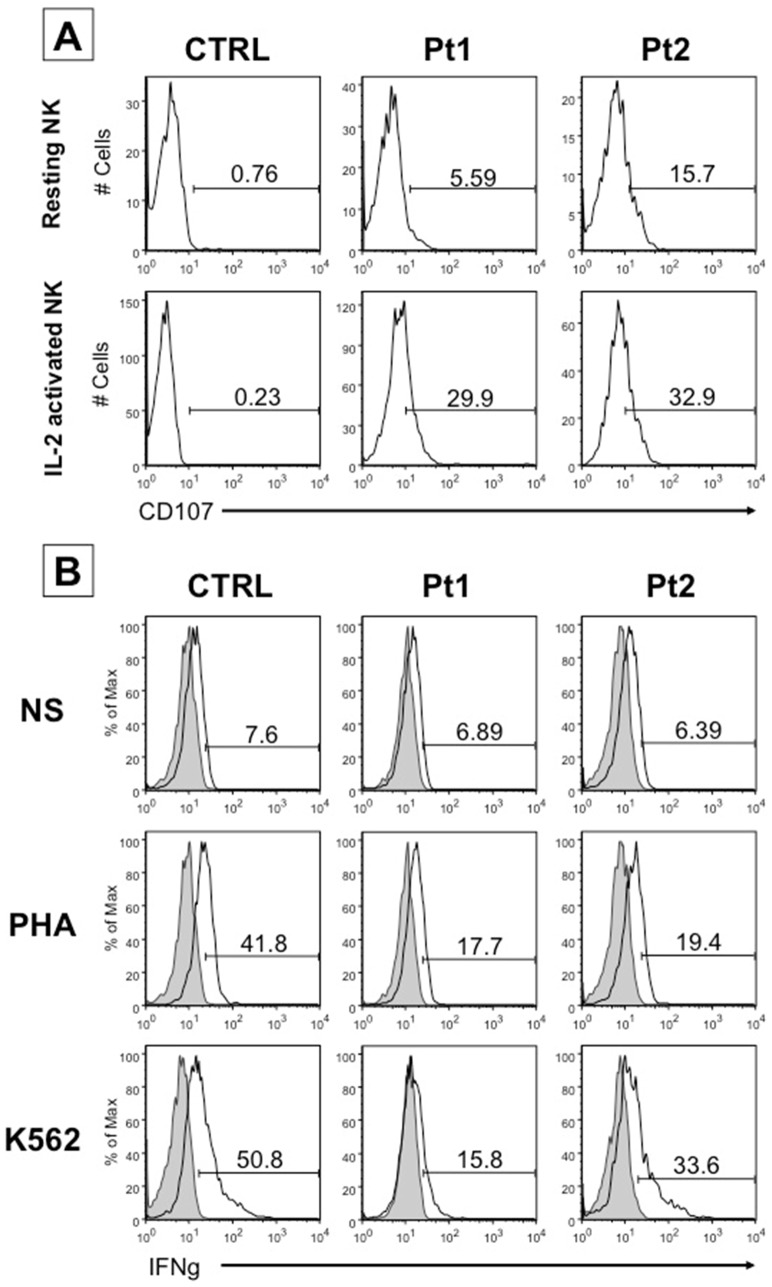
CD107a expression and IFN-γ production by NK cells of HPS2 patients. **A**, surface expression of CD107a in resting (upper panels) and IL-2 activated NK cells (lower panels) in a representative healthy donor (CTRL) of six distinct subjects analyzed and in HPS2 patients (Pt1 and Pt2). In each plot the bar defines the percentage of cells that express CD107a. **B**, IFN-γ–producing IL-2 NK cells were analyzed by flow cytometry. The percentage of IFN-γ producing CD56^+^ NK cells from a representative healthy donor (CTRL) of six distinct subjects analyzed and from patients (Pt1 and Pt2) is shown in un-stimulated conditions (**medium**), stimulated by PHA (**PHA**) and stimulated by NK susceptible K562 tumor target cells (**K562**). The percentages shown in the panels A and B are representative of an experiment repeated three times.

The expression of the degranulation marker CD107a on surface of resting and IL-2-activated NK cells can be induced during exocytosis of lytic granules upon engagement by specific ligands expressed on target cells. In control subjects, CD107a is detectable at very low levels (<1%) on either resting and IL-2 activated NK cells, in the absence of co-culture with tumor target cells. On the contrary, NK cells from HPS2 patients spontaneously expressed CD107a on the surface, at significantly higher levels either at basal level (6% for Pt1 and 16% for Pt2) and upon IL-2 activation (30% for Pt1 and 33% for Pt2) despite the absence of target cells (Figure 4A).

### IFN-γ production and NK-cytotoxic activity are impaired in NK cells from HPS2 patients

Activated NK cells release several cytokines that modulate other effector functions of the immune system. We analyzed the production of IFN-γ, by intracellular staining, in three settings: in IL-2 activated NK cells, after stimulation with phytohemagglutinin (PHA) or after co-culture with the tumor target cells K562 at E/T ratio 1∶1. We observed that IFN-γ was expressed by 17–19% of PHA-activated NK cells from HPS2 patients and by 42% of IL-2 activated NK cells from a representative control subject (Figure 4B, intermediate panels). In addition, incubation of NK cells with the target cell line K562 leads to higher IFN-γ expression in cells from a representative control subject (50%) than from HPS2 patients (16% for Pt1 and 34% for Pt2) (Figure 4B, lower panels), suggesting an impaired production of the cytokine after stimulation.

**Figure 5:**
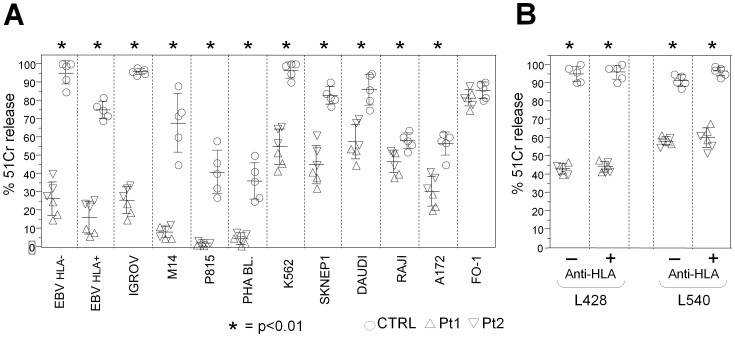
Impairment of cytolytic activity of IL-2–stimulated NK cells in HPS2 patients. **A**, Purified polyclonal IL-2–activated NK cells, derived from Pt1 (triangle pointing up) or Pt2 (triangle pointing down) or 5 distinct healthy donors (circle), were tested against NK susceptible target cells (E/T ratio 10∶1): HLA- LCL 721.221 EBV-lymphoblastoid cell lines, HLA+ ALINA EBV-lymphoblastoid cell lines, IGROV ovarian carcinoma, M14 human melanoma, P815 murine mastocytoma, allogeneic PHA blasts, K562 erythroleukemia, SKNEP1 kidney carcinoma, Daudi and Raji Burkitt's lymphoma, A172 human glioblastoma and FO-1 human melanoma. **B**, Purified polyclonal IL-2-activated NK cells, derived from Pt1 (triangle pointing up), Pt2 (triangle pointing down) or 5 distinct healthy donors (circle), were tested against the Hodgkin's lymphoma derived L428 (HLA-) and L540 (HLA+) cell lines, either in the absence (−) or in the presence (+) of anti-HLA class I mAb at E/T ratio 10∶1. The results shown represent the combination of three independent experiments. Each value represents the mean ± SD of 5 replicates. *  = p<0.01.

We have previously shown an important reduction of cytolytic activity of NK cells in HPS2

 patients [12]. Since both HPS2 siblings developed NLPHL, cytolytic activity of polyclonal IL-2-activated NK cells was evaluated against human and murine tumor cell lines, including Hodgkin's lymphoma cell lines (L428 and L540 cell lines, HLA class I negative and positive, respectively). We detected a striking absolute and/or relative defect of NK cytotoxic activity against B-EBV lymphoblasts, IGROV and M14 human cell lines, P815 murine cell line and PHA activated lymphoblasts, with p<0.01 when compared with control NK cells (Figure 5 A column 1–6). K562, SKNEP1, DAUDI, RAJI and A172 human cell lines showed less impaired, although still significantly lower (p<0.01), cytotoxic activity of HPS2 NK cells when compared to that of normal subjects (Figure 5 A column 7–11). In contrast, the cell line FO-1 was equally susceptible to lysis by both HPS2 and normal NK cells (Figure 5A column 12).

In order to evaluate the role of KIR receptors in NK cytotoxicity against Hodgkin Lymphoma cell lines, HLA class I masking was performed by addition of anti-HLA class I mAbs to the culture before the cytotoxicity assay against L540 and L428 cell lines. NK cells from healthy donors were able to kill both target cell lines at maximal levels (Figure 5B). On the contrary, HPS2 NK cells displayed poor cytolytic activity against both Hodgkin's lymphoma cell lines without any increase after HLA class I masking suggesting that this reduction is not related to the involvement of HLA class I specific inhibitory receptors (Figure 5B).

Analysis of NK activating receptors NKp46, NKp30, NKp44, NKG2D and CD244 (2B4) did not reveal abnormal expression patterns in NK cells of HPS2 patients [12]. This suggests that the heterogeneity of cytolytic activity might be related to the variable surface expression of the specific ligands for NK activating receptors by tumor cells. In order to define the susceptibility of target cell lines to NK cells we performed a further cytotoxicity assay after masking activating NK receptors with different specific monoclonal antibodies directed against NK receptors (data not shown). We observed that tumor target cell lines that express the ligands for NKG2D and NKp30 such as FO-1 and RAJI, were more susceptible to HPS2 NK cells than other tumor cell lines mainly expressing the ligands for NKp46 (M14, P815, B-EBV lymphoblastoid cell lines) that were not killed by patients' NK cells.

### iNKT cells are undetectable in HPS2 patients

**Figure 6:**
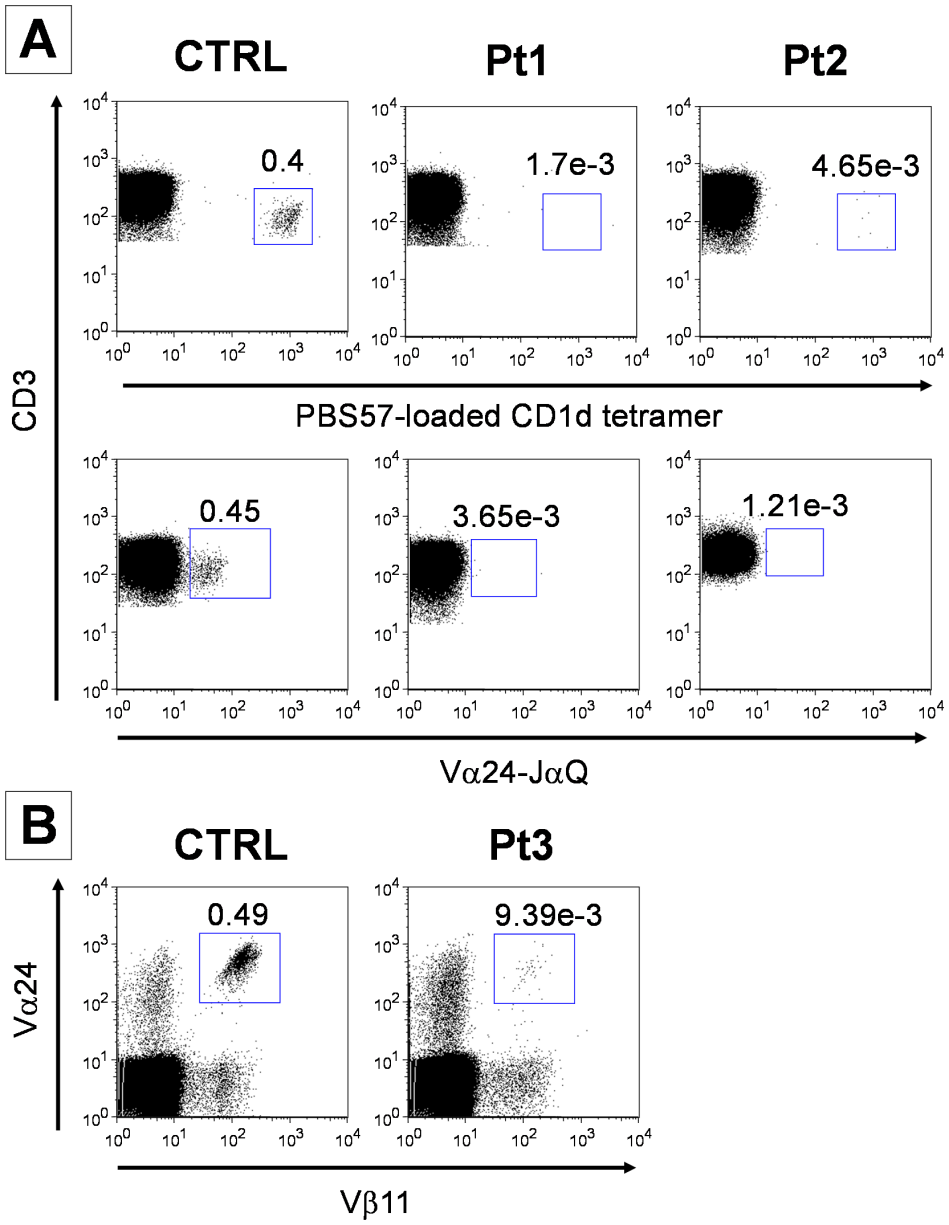
Quantification of iNKT cells in HSP2 patients. Flow cytometric quantification of iNKT cells in isolated PBMC from HPS2 patients and two control subjects concomitantly stained. **A**, The proportion of iNKT cells was calculated on the CD3+ population (at least 250,000 events acquired) contained in the lymphogate and is reported for each dot plot. Staining with PE-conjugated PBS57-loaded CD1d tetramer or, with FITC-conjugated antibody against the Vα24-JaQ invariant chain are presented in the upper and lower panel, respectively. **B**, iNKT cells were counted as double positive for FITC-conjugated antibody against Vα24 and PE-conjugated antibody against Vβ11 in a CD3+ gate comprising at least one million of events. The panel is representative of three separate analyses performed in a 18-month period.

In order to evaluate the number of circulating iNKT cells in HPS2, we have stained PBMC from both patients and 12 healthy donors with an appropriate mixture of mAbs against surface markers (CD3, CD16 and CD56) followed by the incubation with PE-conjugated PBS57-loaded or PBS57-unloaded CD1d tetramer. Despite the large number of CD3^+^ lymphocytes analyzed (at least 250,000 events acquired), Pt1 and Pt2 cells stained with the loaded tetramer displayed a number of PE-positive events similar to the ones obtained staining with the unloaded tetramer (Figure 6A, upper panels). The same result was obtained when patients PBMC were stained with a commercial antibody directed against the CDR3 region of the invariant chain (Vα24i), specifically expressed by iNKT cells (Figure 6A lower panels). For this reason patient's iNKT cell counts had to be considered below the detection limit of the technique. Evaluation of circulating iNKT was performed in three separate occasions in a 18-month period with an average of 3.16e-3 and 2.55e-3 for Pt1 and Pt2 respectively, constantly below the range reported for normal subjects.

Quantification of iNKT cells in an additional HPS2 patient (Pt3) by staining with the combination of antibodies against the αβTCR chains specifically expressed by iNKT cells (Vα24 TCR and Vβ11 TCR) showed a similar reduction of iNKT cells (Figure 6B).

## Discussion

In this study we report the occurrence of nodular lymphocyte predominance type Hodgkin lymphoma (NLPHL) in two young siblings affected by Hermansky Pudlak type 2 syndrome (HPS2). By analyzing peripheral blood immune cells we found that NK and iNKT cells from HPS2 patients are significantly impaired in their number and function including tumor cell killing activity. On histology, NLPHL from HPS2 patients show an increased number of Prf+ CD8+ CTLs and at lower extent CD56^+^CD3^−^ NK cells within the tumor nodules. While, CD3+CD8+ T cells from HPS2 patient presented a normal content of perforin. Taken together, these results suggest that HPS2 NLPHL show an increased number of Prf^+^CD8^+^ CTLs within the tumor nodules; this might reflect a defect in the release of this granules-associated protein by the CTL fraction.

Different forms of lymphoproliferative disorders (LPD) are observed in primary immune deficiency (PID) patients and are commonly related to EBV infection [32]. NLPHL occurring in HPS2 patients, similarly to sporadic NLPHL, are probably not related to EBV infection since EBV antigens were not detected in the tumor cells. This suggests that impairment of T-cell-mediated immune surveillance to EBV is not involved in the mechanism of HPS2-associated lymphomagenesis [33]. NLPHL represents a minor fraction of HL and derives from the neoplastic transformation of germinal centre B cells at the centroblastic stage. Familial NLPHL are rarely observed in immune competent subjects [34]–[37], but they are commonly seen in patients suffering from autoimmune lymphoproliferative syndrome (ALPS) [7], [8], a disorder of apoptosis in which the inability of lymphocytes to undergo programmed cell death leads to lymphadenopathy, hypersplenism, and autoimmune cytopenias increasing the risk of developing Hodgkin (HL) and non-Hodgkin lymphoma (NHL).

The development of NLPHL in the two HPS2 siblings suggests a possible involvement of effector functions of multiple cell types including NK, iNKT and CTL in lymphoma development. In this study we found an abnormal distribution of NK subsets in the peripheral blood of HPS2 patients. Specifically, HPS2 patients show a significant reduction of CD56^bright^ CD16^−^ KIR^−^ NKG2A^+^CCR7^+^ subset. Recent reports support the hypothesis of unidirectional differentiation of CD56^dim^ from CD56^bright^, suggesting that the recurrent viral infections might lead to depletion of CD56^bright^ CD16^−^ KIR^−^ NKG2A^+^CCR7^+^ NK cells [13], [26], [28]. Within the CD56^dim^ subset, a more differentiated phenotype is highlighted by the simultaneous expression of CD57 and of KIR, CD16 and intense intracytoplasmic Prf. Remarkably, CD56^dim^ NK cells from HPS2 patients expressed CD57 molecule and KIRs repertoire, suggesting that these cells have completed their maturation in spite of reduced intracellular Prf content. Moreover, various authors demonstrated that recurrent viral infection can cause an increase of CD57^+^ NK cells reflecting differentiation and expansion of a human memory NK cells subset as probably occurred in Pt1 [38].

It is commonly accepted that CD56^bright^ NK cells are the main source of cytokine production, while CD56^dim^ NK cells are mostly responsible for cytolytic activity and tumor target cell killing. However, recent evidences indicate that also early IFN-γ production is a functional property of CD56^dim^ NK cells after engagement of activating receptors [39]. Notably, CD56^+^CD3^−^ NK cells from HPS2 patients show an important cytolytic defect combined to a reduced production of IFN-γ after engagement of activating receptors. With the exception of FO-1, NK cells from HPS2 patients fail to properly recognize and kill a large series of tumor cell lines including HL cell lines. Analysis of NK activating receptors by polyclonal IL-2 activated NK cells of both HPS2 patients did not reveal abnormal expression. This observation suggests that a reduced surface density expression of activating NK receptor ligands on tumor cell lines, not involving the whole repertoire of activating NK receptors, might account for the severe cytotoxic defect of HPS2 NK cells. Remarkably, despite HPS2 patients have defective cytotoxic activity, the risk of these patients to develop hemophagocytic lympohistiocytosis (HLH) is very low since only a single HPS2 patient developing HLH has been reported [30]. The observation that HPS2 patients show reduced production of IFN-γ after engagement of activating receptors might be important to understand the differences between HPS2 and other genetic conditions characterized by impairment of NK/CTL cytotoxic activity [40].

Previous observations showed that cell-surface expression of CD63 was increased in fibroblasts and CTL of HPS2 patients suggesting that lack of AP-3 complex results in change in the steady state distribution of the membrane protein between intracellular vesicles and the plasma membrane. We reported a remarkable increase of CD63 expression on the cell surface of neutrophils and a severe defect of neutrophil elastase expression in the cytoplasm as compared with control subjects [12]. This observation led us to speculate that lack of AP-3 prevents normal expression and correct sorting of neutrophil elastase in azurophil granules of myeloid progenitors. In this study we have shown that unstimulated and IL-2 -stimulated NK cells from both HPS2 patients expressed increased baseline levels of CD107a and CD63, suggesting that this misrouting of lysosomal proteins to the cell membrane could likely contribute to the observed cytotoxic defect and impaired degranulation of cytolitic proteins. It is noteworthy that Enders et al. demonstrated an increased baseline expression of lysosomal marker proteins such as CD63 and CD107a on resting CD3^+^CD8^+^T cells from HPS2 patients, but their elevated expression levels did not further increase on surface despite functional activation of the cells [30].

In 2003 Clark et al. [31] showed that AP-3 deficiency leads to a diminished CTL-mediated cytotoxicity dependent on a defective lytic granules movement along the microtubules to the microtubule organizing center (MTOC). As a result, AP-3 deficient CTLs from HSP2 patients showed enlarged lytic granules with an abnormal cytoplasmic distribution on microscopy. Noteworthy, we found a high number of Prf+ CTL in NLPHL from HPS2 patients, suggesting intracellular retention of this protein in tumor-associated CTL.

In addition to the numerous tumour-infiltrating CTL, also NK cells play an important role in the immune surveillance against lymphomas. In fact, NK cells are usually seen as sentinel cells that can interfere with lymphoma growth at the early stages of development while the CTL might infiltrate the tumor at the later times [41].

Another remarkable finding in HPS2 patients is represented by the profound depletion of iNKT cells. This is in line with the evidence that AP3-deficient mice have a reduced iNKT cell population, suggesting a developmental defect possibly associated with impaired intracellular trafficking of CD1d [42]. Alternatively, the reduction of iNKT cells in HPS2 patients might be a secondary phenomenon due to repetitive viral infections [43].

Among PID, a striking defect of iNKT cells is observed in X-linked lymphoproliferative disease (XLP). Most XLP cases are caused by germline mutations in the SH2D1A gene, which encodes the adaptor molecule Signaling Lymphocytic Activation Molecule (SLAM)-associated protein (SAP) [44]. Loss of SAP expression in XLP patients and mice impairs NK and CD8+ T cells cytotoxicity, T cell cytokine production, activation-induced cell death, and iNKT cell development. Although XLP patients typically develop EBV-associated non Hodgkin B cell lymphomas [45], recent studies suggest that T cells restricted to non-classical MHC Class-I molecules are important for immune surveillance of hematological malignancies [46]. In particular, iNKT cells, that are CD1d-restricted cells, can induce cell death of chronic lymphocytic leukemia cells after stimulation with alphaGalCer [47], and can trigger secondary anti-lymphoma response in murine models of lymphoma [48]. Significantly, CD1d is expressed by neoplastic cells of HL and iNKT cells have been detected in cell suspensions of HL clinical samples [46]. These data indicate a potential contribution of iNKT cells to NLPHL development.

In summary, this study reports (for the first time) the occurrence of NLPHL in two siblings with HPS2. The set of abnormalities observed in different immune cell compartments point toward HPS2-associated NLPHL as an additional model to understand the role of the immune surveillance in B-cell lymphomas. The availability of mice with distinct mutations in the AP3B1 gene and mimicking HPS2 will be instrumental in clarifying the role of the AP-3 complex in abnormal lymphoid cell proliferation.
